# Proteome study of cutaneous lupus erythematosus (CLE) and dermatomyositis skin lesions reveals IL-16 is differentially upregulated in CLE

**DOI:** 10.1186/s13075-021-02511-0

**Published:** 2021-04-30

**Authors:** Timothy B. Niewold, Alexander Meves, Julia S. Lehman, Karin Popovic-Silwerfeldt, Aliisa Häyry, Therese Söderlund-Matell, Cristine M. Charlesworth, Benjamin Madden, Ingrid E. Lundberg, Marie Wahren-Herlenius, Elisabet Svenungsson, Vilija Oke

**Affiliations:** 1 Colton Center for Autoimmunity, New York University School of Medicine, New York, NY USA; 2 Department of Dermatology, Mayo Clinic, Rochester, MN USA; 3 Department of Laboratory Medicine and Pathology, Mayo Clinic, Rochester, MN USA; 4 Dermatology Clinic, Department of Clinical Sciences at Danderyd Hospital, Karolinska Institutet, Stockholm, Sweden; 5 Division of Rheumatology, Department of Medicine, Karolinska Institutet, Stockholm, Sweden; 6 Rheumatology, Karolinska University Hospital, Stockholm, Sweden; 7 Mayo Clinic Medical Genome Facility - Proteomics Core, Rochester, MN USA; 8 Center for Rheumatology, Academic Specialist Center, Stockholm Healthcare Services, Stockholm, Sweden

**Keywords:** Cutaneous lupus erythematosus, Systemic lupus erythematosus, Dermatomyositis, Cytokine, Interferon, Complement

## Abstract

**Background:**

The objective of the study was to explore the disease pathways activated in the inflammatory foci of skin lesions in cutaneous lupus erythematosus (CLE) and dermatomyositis (DM).

**Methods:**

Skin biopsies acquired from active CLE and DM lesions, patient (PC), and also healthy controls (HC) were investigated. Biopsy sections were examined by a pathologist, inflammatory foci were laser micro-dissected and captured, and proteins within captured tissue were detected in an unbiased manner by mass spectrometry. Protein pathway analysis was performed by the string-db.org platform. Findings of interest were confirmed by immunohistochemistry (IHC).

**Results:**

Proteome investigation identified abundant expression of interferon-regulated proteins (IRP) as a common feature of CLE and DM. Interleukin (IL)-16 was the only abundant cytokine differentially expressed in CLE compared to DM. Caspase-3, an enzyme that cleaves IL-16 into its active form, was detected in low levels. Significantly higher proportion of IL-16- and caspase-3-positive cells was identified in CLE lesions in comparison with DM, PC, and HC. Proteomic results indicate more abundant complement deposition in CLE skin lesions.

**Conclusions:**

Using unbiased mass spectrometry investigation of CLE and DM inflammatory infiltrates, we confirmed that high IRP expression is a common feature of both CLE and DM, while IL-16 is the only differentially expressed cytokine in CLE. IHC confirmed high expression of IL-16 and caspase-3 in CLE. Our novel molecular findings indicate that IL-16 detection could be useful in differential diagnostics between the two conditions that can display similar histopathological appearance. IL-16 could be of interest as a future therapeutic target for CLE.

## Background

Systemic lupus erythematosus (SLE) is an autoimmune disease with a range of clinical manifestations including systemic inflammation, circulating autoantibodies against nuclear antigens and other intracellular molecules, and frequent involvement of the skin, joints, and also renal and hematological systems. If not treated, autoimmune inflammation may lead to organ damage, and in severe cases, vital organ involvement can be life-threatening. Cutaneous lupus erythematosus (CLE) is one of the specific manifestations of SLE but also may develop in patients without systemic disease. Still, some of the CLE patients will later progress to SLE [1, 2]. The autoimmune inflammation observed in SLE and CLE is a result of activated inflammatory pathways within both the innate and adaptive immune systems, and interferons (IFNs) are thought to be the key mediators [3].

Dermatomyositis (DM) is another systemic autoimmune disease, primarily affecting the muscles and skin. DM can also affect the lungs and myocardium among other systems, and the vital organ involvement in this disease can also be lethal [4]. In DM, anti-nuclear antibodies are common, as are autoantibodies directed against tRNA synthetases [5].

Genome-wide association studies revealed that many genes within the IFN pathway could determine susceptibility to either cutaneous or systemic LE and also dermatomyositis [6–8]. Upregulation of IFN-regulated proteins is observed in both peripheral circulation and at the target organ inflammation in CLE, SLE, and DM [7–9]. The exact reason and molecular mechanism of the activation of the IFN system are not known. Disturbances in cell death, clearance of unviable cell debris, and activation and deposition of complement components are known to occur in skin lesions in CLE and in muscle fibers in DM. These factors could activate the IFN pathway via pattern recognition receptors and further lead to autoimmune inflammation [9, 10].

The typical histopathologic patterns of CLE and DM skin lesions are surprisingly similar [4]. Specifically, the microscopic patterns include interface dermatitis, apoptosis of keratinocytes, perivascular and perifollicular lymphohistiocytic inflammation, and increased dermal mucin. Despite the similarities in skin biopsy, CLE and DM are very different clinically in respect to the distribution of rash and the organ system involvement. Interleukin (IL)-18 has been implicated as a cytokine differentially expressed in DM, but not CLE lesions [9]. It is not clear what other molecular differences underlie the clinical differences and histopathologic similarities between CLE and DM.

In the current study, we investigated the total proteome of laser micro-excised inflammatory foci of CLE and DM lesions in an unbiased manner in order to identify the differential disease-specific molecular pathways.

## Methods

### Patients

Patients with cutaneous lupus erythematosus (CLE) rash (*n* = 13) were included at disease exacerbation at either the Rheumatology Department at Karolinska University Hospital (KS) or the Dermatology Department at Danderyds Hospital (DS), Stockholm. Patients with dermatomyositis were recruited at diagnosis or disease exacerbation at KS (*n* = 7). All subjects gave informed written consent at inclusion. The study was approved by the Swedish Ethical Review Agency and was conducted in compliance with the Helsinki Declaration.

At inclusion, each patient was examined by a physician. The activity of the skin rash in CLE patients was scored using the Cutaneous Lupus Disease Area and Severity Index (CLASI) [10]. The activity of DM rash was assessed using the Cutaneous Dermatomyositis Disease Area and Severity Index (CDASI) [11]. SLE activity was assessed using systemic lupus assessment measure (SLAM) and its definitions [12]. All patients had their CLE diagnosis confirmed by a dermatopathologist, and the majority of the patients had a diagnosis of SLE at inclusion. The patients with SLE diagnosis met the ACR criteria for SLE [13]. The diagnosis of dermatomyositis was based on cutaneous and muscular histopathologic features, as well as serological and blood chemistry tests indicating muscle damage by elevation of muscle enzymes [14]. Biopsies from active skin lesions (CLE or DM) distributed on the extremities, trunk, or scalp were acquired in local anesthesia using the punch technique. Biopsies from uninvolved skin were acquired from the UV-non-exposed buttock skin from 5 of the included CLE patients (PC). Five healthy controls (HC) were recruited at the Dermatology Department, DS, while undergoing excision of dysplastic naevus (extremities or trunk), where extra skin “tags” were needed to be excised due to medical necessity in order to achieve cosmetically acceptable would closure. Only naevi and dysplasia-free parts of the samples were used.

The demographic and clinical characteristics of the cohort are presented in Table 1. Information on autoantibodies and medications is presented in Additional Table 1.

**Table 1:** Characteristics of the cohort

	CLE, *n* = 13	DM, *n* = 7	Healthy controls, *n* = 5	CLE uninvolved, *n* = 5
Cases included in proteomics	5	5	5	0
Age, M (SD)	59 (18)	52 (12)	66 (21)	
Sex, female	13	5	3	
CLE/SLE	13/10	–		
DM/amyopathic DM	–	6/1		
Organ involvement at inclusion				
Active rash	13	7		
Active arthritis	5	0		
Active nephritis	2	0		
Active CNS	2	0		
Active myositis	0	6		
Paramalignant disease	0	3		
Cutaneous activity indices				
CLASI (available in 10) MD (IQR)	7.5 (6–10.25)	–		
CDASI (available in 6), MD (IQR)	–	18 (12.25–20.75)		

*CNS* central nervous system, *CLASI* Cutaneous Lupus Disease Area and Severity Index, *CDASI* Cutaneous Dermatomyositis Disease Area and Severity Index, *MD* median, *IQR* interquartile range

### Laser capture microdissection and proteomics

Skin biopsies were acquired using the 4-mm punch method and fixed in formalin, thereafter embedded in paraffin and sectioned. The skin sections were examined by an experienced dermatopathologist (Dr. J. Lehman) who confirmed the histopathological features of CLE and DM. Laser capture microdissection-assisted liquid chromatography-based tandem mass spectrometry was performed as follows. Ten-micrometer-thick sections of formalin-fixed, paraffin-embedded skin specimens were mounted onto polyethylene naphtalate membrane slides and stained with Congo Red stain (Sigma-Aldrich, St. Louis, MO) for microanatomic visualization. Laser capture microdissection was used to isolate the inflammatory foci within the dermis. A total of 1,000,000μm^2^ of tissue per case was dissected; tissue elements were collected in digestion buffer in 0.5-ml caps. After processing and digest, each sample was analyzed by nano-LC tandem mass spectrometry at Mayo Clinic Medical Genome Facility - Proteomics Core.

### Immunohistochemistry (IHC)

In order to verify the proteomics findings, we employed the IHC technique. Sequential skin biopsies were processed and stained for IL-16 and caspase-3. First, the sections were heated overnight at 60 °C, thereafter deparaffinized in xylene and washed in ethanol. Antigen retrieval was performed by boiling the sections for 30 min in 10 mM citrate buffer (pH = 6). Permeabilization was performed by washing in 1 M PBS buffer, pH 7.4, containing 0.1% saponin (PBS-S). Non-specific tissue binding was blocked by incubating the sections in 3% hydrogen peroxide (H_2_O_2_) in PBS-S for 20–30 min, which was followed by blocking using an Avidin/Biotin blocking kit (SP-2001, Vector Laboratories) and thereafter by incubating in 2% bovine serum albumin (BSA) and 2% milk in PBS-S. The rabbit polyclonal anti-human IL-16 (HPA018467, Sigma) and anti-caspase-3 antibodies (HPA018467, Sigma) were diluted 1:500 and 1:1250, respectively, in 1% BSA and 1% milk in PBS-S solution, applied to the sections and incubated overnight at room temperature. For control stains, we either omitted the primary antibody or the primary antibodies were replaced by rabbit immunoglobulin fraction (X0936, Dako, 1:1000). Next, the sections were washed in the buffer 5 min, 3 times, and blocked with 2% normal goat serum (NGS) in 20 min. The secondary antibody, a biotinylated polyclonal goat anti-rabbit IgG (B8895, Sigma, 1:500), was applied on the sections in a buffer containing 1% NGS and incubated for 60 min. The sections were developed using extravidin peroxidase (E2886, Sigma, 1:400) and DAB-HRP substrate kit (SK-4100, Vector Lab). The glasses were counterstained with hematoxylin and mounted with Mountex (Histolab).

### Data collection

The sections were visualized using a Leica DMR XA2 light microscope. Semiquantitative assessment of the staining was performed in a blinded manner by two investigators (AH and TSM) and supervised in case of discrepancy by a third investigator (VO). Images in ×20 amplification were scored and obtained of at least two representative infiltrated upper-dermal areas in each LE and DM section. At least one representative stained dermal area in each control skin section was photographed in a similar manner. Standardized visual settings were used throughout the photographing process.

The total number of present/infiltrating cells in each image was counted using a standardized counting method. Each image file was opened in the QuPath v0.1.2 digital pathology image analysis software, and all stained cells were evaluated individually and marked manually in a systematic manner using the counting tool [15]. The visual settings of the computer screen in use were standardized throughout the process. Positive staining was defined as brown cytoplasmic staining. Infiltrating or tissue cell was defined as a cell located in the extracellular matrix of dermal connective tissue outside of the dermal appendages and blood vessels.

### Data analysis and statistics

All data were analyzed in the following groups: CLE, DM, HC, and for IHC, uninvolved skin of CLE patients was also included (PC).

**Fig. 1:**
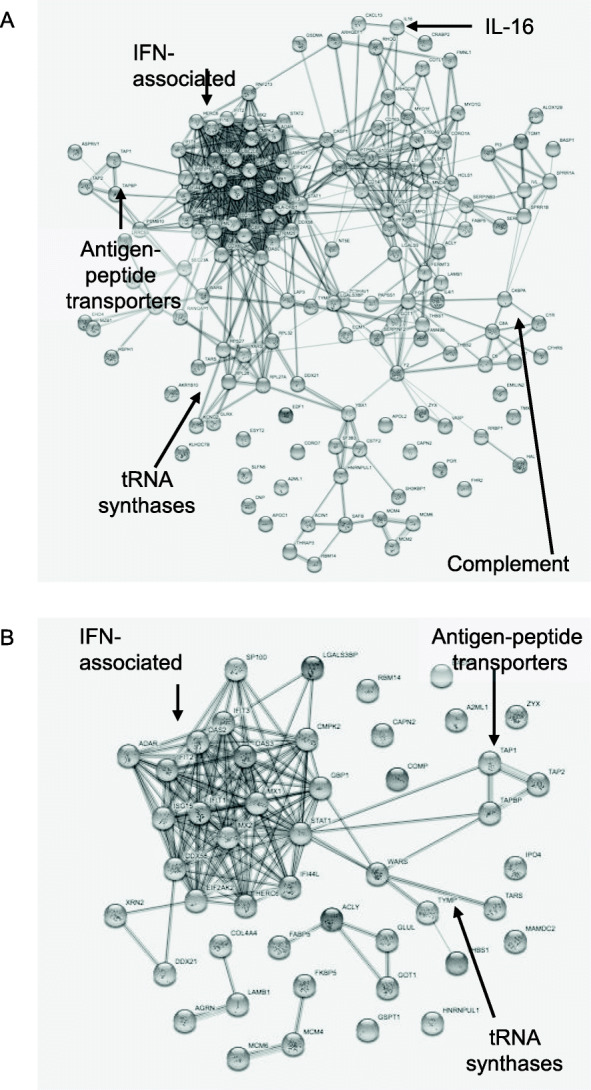
The protein network of the upregulated proteins (5-fold or more) in cutaneous lupus erythematosus (CLE) vs healthy controls (**a**) and dermatomyositis (DM) vs controls (**b**), analysis performed by string-db.org online platform. The significant protein networks are indicated by arrows

For the mass spectroscopy data, total spectral counts were used to calculate the fold change ratios between the patient groups and controls. We investigated the proteins that were differentially expressed (> 5-fold expression; *p* < 0.05; false discovery rate < 1%) between each comparison group. These protein lists were entered into the string-db.org program for pathway analysis and visualization (Fig. 1).

For the immunohistochemistry data, the total number of the present and positively stained cells was calculated for each section and protein. The proportion of stained cells out of the total dermal cell number was calculated. The percentages of the stained cells were compared among the groups using the Wilcoxon non-parametric or Mann-Whitney tests as appropriate; *p* values < 0.05 were considered significant. Microsoft Excel 365 and JMP13 software (SAS Institute, Carey, NC, USA) were used for all statistical testing.

## Results

### Total protein analysis of inflammatory foci in CLE and DM

We analyzed the total proteome of excised inflammatory foci of CLE and DM lesions in order to identify the upregulated proteins of major importance. The unbiased total proteome analysis identified over 2000 proteins that were detected at a level of at least 5 copies per sample. Proteins in lower abundance were considered to have higher variability and be hits of secondary interest and are not discussed further (Additional Table 2). Ratios of total spectral counts for these 2000 proteins were compared among three groups: CLE patients, DM patients, and HC.

Proteins upregulated 5-fold or more in comparison with HC were regarded as interesting hits and included in further analyses (Fig. 1 and Additional Table 2).

### Interleukin-16 was identified as a unique highly abundant cytokine in the CLE lesions

The proteomic analysis identified IL-16 as a single top-upregulated cytokine in the inflammatory foci of CLE. Only a limited amount of copies of IL-16 were detected in DM and in HC (Table 2). IL-16 is an intracellular protein. In order for it to become functional, upon T cell stimulation, it must be cleaved by caspase-3. Caspase-3 was detected in CLE lesions by proteomic analysis, but at low levels (fewer than 5 peptides).

**Table 2:** Comparison of the most abundantly expressed proteins in the inflammatory foci of CLE and DM skin lesions

Numbers of proteins	CLE/HC	DM/HC	CLE/DM
Interleukin-16 (IL-16) (x fold)	12	1	12
IFN regulated and associated proteins, high in both LE and DM (x fold)			
IFIT1	71	57	1.2
IFIT3	70	46	1.3
DDX58	48	33	1.5
ISG15	48	30	1.6
OAS2	36	21	1.7
MX1	31	16	2
MX2	29	14	2
STAT1	29	16	1.9
OAS3	28	33	0.83
EIF2AK2	23	15	1.5
IFI44L	18	18	1
IFIT2	13	11	1.2
DDX21	6	6.5	1
IFI16	6	4.5	1.1
ADAR	7.3	5	1.4
IFN-regulated and associated proteins, high in LE but low in DM (x fold)			
MNDA	31	Low*	31
PTPN6	24	Low*	24
DDX60	18	Low*	18
STAT2	14	Low*	3.8
ZC3HAV1	14	Low*	7
OASL	14	Low*	14
IFI44	10	Low*	1.6
IFIT5	8	Low*	2
OAS1	8	Low*	8
Antigen processing and transport (x fold)			
TAP1	18.5	10	1.9
TAP2	24	19	1.3
TAPBP	5.4	1.1	
Aminoacyl-tRNA synthases and other enzymes (x fold)			
WARS	90	55	1.6
TARS	13	10	1.3
TYMP	9.4	9	1
ACLY	28	9	3
GOT1	8	8	1

*Detected as 5 or fewer peptides, therefore regarded as an unreliable result

### In CLE lesions, high expression of IL-16 coincides with the presence of caspase-3

**Fig. 2:**
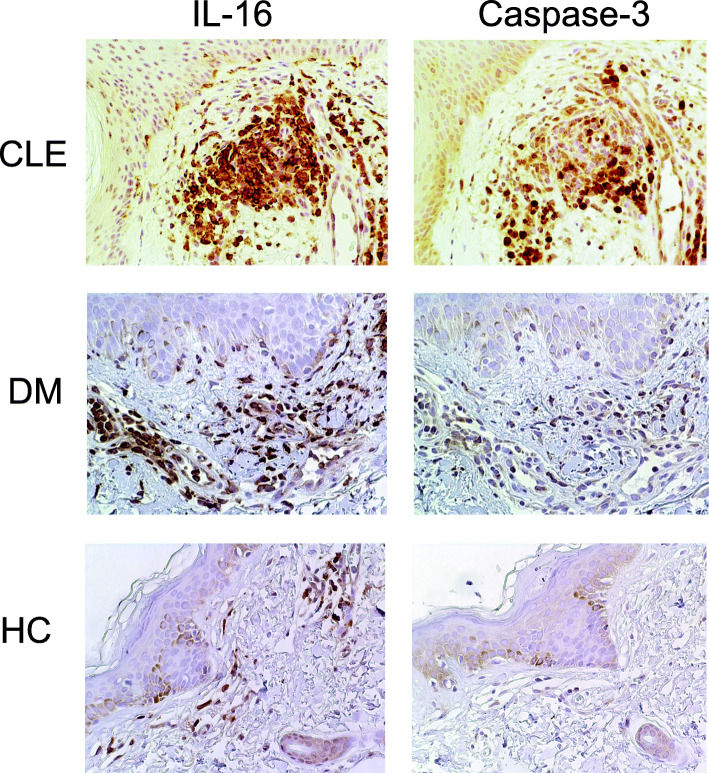
Representative microphotographs of cutaneous expression of IL-16 and caspase-3 as detected by immunohistochemistry in skin biopsies of cutaneous lupus erythematosus (CLE), dermatomyositis (DM), and healthy controls (HC) (×20 magnification)

In order to verify our observation, we performed IHC staining for IL-16 on CLE (*n* = 13) and DM (*n* = 7) skin lesions, unaffected skin of CLE patients (*n* = 5), and HC skin (*n* = 5). We confirmed that a high proportion of infiltrating cells in CLE was positive for IL-16 (Fig. 2).

**Fig. 3:**
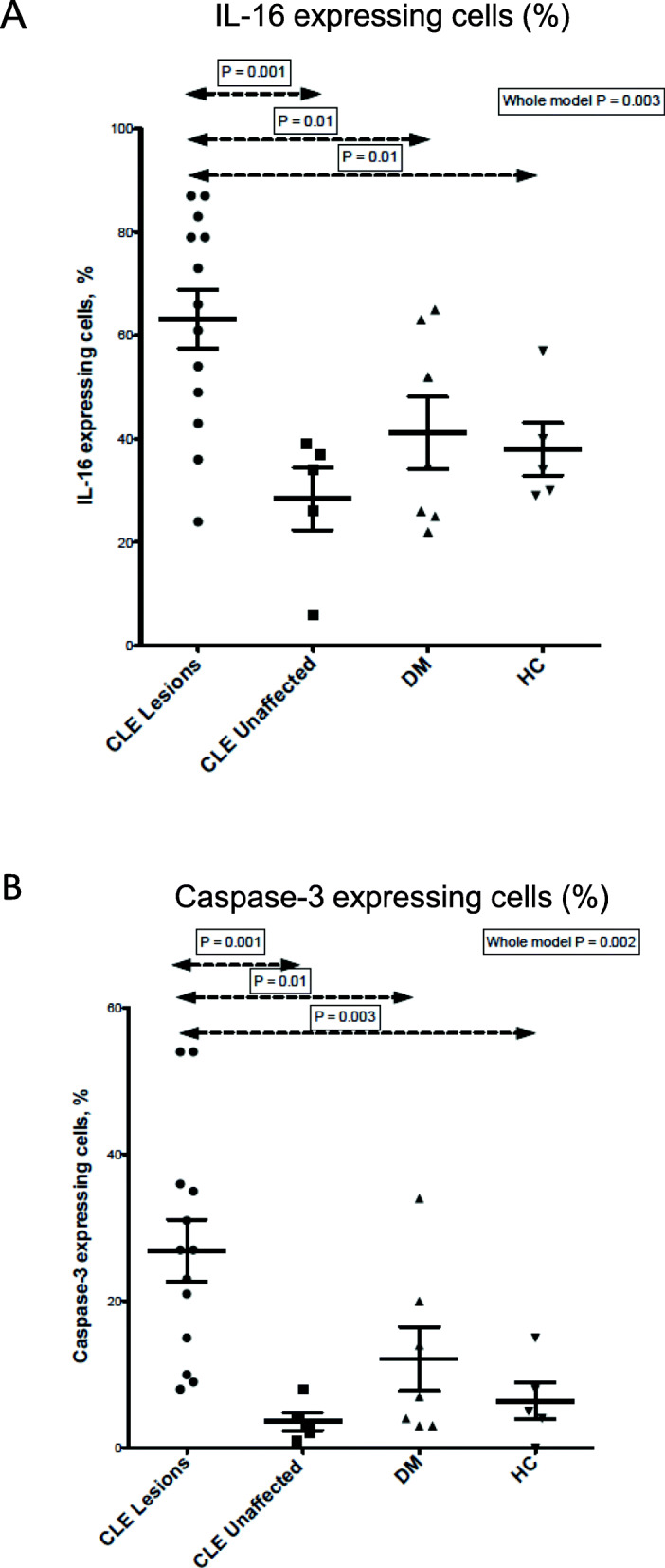
Semiquantitative analysis of IL-16 (**a**) and caspase-3 (**b**) expression in cutaneous lupus erythematosus (CLE), dermatomyositis (DM), CLE uninvolved, and HC skin. Statistical analysis (non-parametric Wilcoxon test) was performed using estimated proportion (%) of positive vs total amount of infiltrating or present cells per high-power field (HPF) at × 25 magnification. The Mann-Whitney test was used for comparisons between each group

Semiquantitative analysis of IHC staining revealed that approximately 66% (interquartile ratio (IQR) 46–81) of the cells comprising the inflammatory foci in CLE expressed IL-16 (Figs. 2 and 3). IL-16 was detected in both DM, PC, and HC, but the percentage of cells expressing IL-16 was significantly lower (35% (IQR 25–63), 34% (IQR 16–38), and 34% (29–48), respectively) (Figs. 2 and 3, Additional Fig. 1), *p* = 0.01. The cells within the same infiltrates were observed to express caspase-3, the enzyme that cleaves IL-16. Up to 27% (IQR 12.5–35.5) of the cells within CLE infiltrates were positive for caspase-3, while in DM, it was only 7% (IQR 3–20), and even fewer in PC and HC, respectively 3% (IQR 1.5–6) and 5% (IQR 2–22.5) (*p* < 0.001) (Figs. 2 and 3, Additional Figure 1).

There were no significant correlations between the percent of IL-16 or caspase-3-positive cells and CLASI, CDASI activity, or damage indices (data not shown).

### High expression of IFN-regulated proteins in the inflammatory foci of CLE and DM lesions

Interferon (IFN) regulated proteins (IRP) were the most abundant molecules within the inflammatory foci of both CLE and DM, and both conditions were upregulated 20-fold or more. In CLE, the IFN protein signature was stronger and included even more molecular hits than in DM (Table 2 and Fig. 1).

Other molecular pathways activated in both CLE and DM were antigen peptide transporter (TAP) protein network as well as several tRNA synthases and other common enzymes, including aspartate aminotransferase (GOT1) (Table 2).

### Intralesional presence of classical complement activation pathway components is a feature of CLE

In CLE, the proteomic analysis identified high expression of almost all components of the classical complement activation pathway including early and terminal activation proteins, as well as complement regulators and receptors. Only C2 was not detected at all, while C4A and C4B as well as C9 were detected, but upregulated only approximately 1.5-fold, in comparison with HC. These molecules were not detected or detected at a low level in DM or control skin samples (Fig. 1 and Table 3).

**Table 3:** Intralesional expression of the complement components, regulators, and receptors was higher in CLE than in DM

Proteins	CLE/HC	DM/HC	CLE/DM
C1R	9	1	9
C3	1.2	0.6	2
C4BPA	5.5	2	3
C5	4.2	1.3	7
C6	22	Low*	22
C7	18	Low*	18
C8A	6	Low*	12
C8B	4.5	Low*	9
C8G	2	Low*	2.5
CFHR1	2.4	Low*	2.4
CFHR2	9	1	9
CFHR5	14	Low*	14
ITGB2	6	0.6	10

*CLE* cutaneous lupus erythematosus, *DM* dermatomyositis, *C* complement, *C4BPA* complement factor 4 binding protein alpha chain, *CFHR* complement factor H-related protein, *ITGB2* integrin B2

*Detected as 5 or fewer peptides, therefore regarded as unreliable result

## Discussion

In this study, we aimed to explore what proteins are expressed in the inflammatory foci of CLE and DM lesions. Our major finding from tandem mass spectrometry investigation is that IL-16 is the only detectable and highly abundant cytokine in CLE, but not DM lesions. Also, we could confirm high expression of IRP in both conditions.

The study is unique in several aspects. We utilized a novel method—laser capture microdissection which allowed excision of the cells comprising inflammatory foci. The collected tissue was analyzed in an unbiased manner using the mass spectrometry technique. The detected proteins were compared among the groups CLE, DM, and HC. Data analysis using the string-db.org database revealed the involved protein networks, similarities, and also some major differences between the conditions.

Increased levels of circulating IL-16 in SLE patients have been described before, by us and others [16, 17]. Also, upregulation of IL-16 has been observed in psoriasis, systemic sclerosis, inflammatory bowel disease, and several malignancies [17–19]. Our group has previously reported observation that SLE patients with active nephritis or arthritis had high levels of circulating IL-16, while patients with CLE had lower [17]. The presented data indicate that cytokines in the circulation do not necessarily correspond to the molecular processes taking place in the LE target organ, for example, the skin.

Interestingly, other investigators described that in the skin affected by systemic sclerosis, approximately 1/3 of the infiltrating cells express IL-16 [18]. In comparison, in the current study, we found that approximately 2/3 of the infiltrating cells in CLE carry this molecule, while the proportion in DM, PC, and HC was about 1/3, similar to that described systemic sclerosis [18]. The predominant sources of IL-16 are T cells, but also eosinophils, DCs, mast cells, macrophages, and monocytes can also produce IL-16 [12, 13]. IL-16 is generated as a precursor molecule, and when cleaved by caspase-3, two molecules with different functions are generated: N-terminal pro-IL-16 and C-terminal secreted/mature IL-16. Pro-IL-16 molecule functions as a regulator of T cell growth, and a secreted mature IL-16 functions as a CD4 and/or CD9 ligand and facilitate cell motility and activation [12, 13]. It is known, that majority of infiltrating cells in the skin are T cells, and as our results indicate, they carry the IL-16 molecule. We suggest that IL-16 could function as a chemoattractant in the CLE lesions. Also, intranuclear expression of IL-16 is known to impede cell cycle progression and could possibly negatively affect cell growth and regeneration within the skin lesions [19].

Multiple cytokines have been found to be involved and mediate local inflammatory responses in CLE including type I and III IFNs, TNF-α, IL-1β, and HMBG1, as well as the Th17 pathway along with IL-21 [14–17]. Our analysis confirmed that the IFN-regulated protein (IRP) network is the most abundant protein pathway activated in the lesions of both CLE and DM. While this prominence of the IRP network was similar between the two diseases, CLE lesions appeared to have higher expression and diversity of the proteins within the IFN pathway. Interestingly, IFN-α has been demonstrated to be able to increase caspase-3 mRNA levels in activated T cells and induce increased IL-16 secretion, without increasing IL-16 mRNA levels nor inducing cell death [20]. Our findings suggest this interaction should be further explored in CLE.

The transporters associated with antigen processing (TAP-1 and TAP-2) are of importance for normal expression of MHC class I and presentation of intracellular peptides, while TAPBP is a catalyst molecule in the binding of antigen. The defective function of this pathway is implicated in type I autoimmune diabetes mellitus [21]. High expression of these molecules in both CLE and DM suggest that autoantigen presentation could be occurring intracutaneously in inflammatory infiltrates and could be of interest in further investigation.

Aminoacyl-tRNA synthetases (ARS) are another abundant molecular group detected in the lesions of both CLE and DM, but not in the control skin. The detected ARS included tryptophanyl (WARS) and threonyl (TARS) as the most abundant. WARS has been demonstrated to have additional functions in the immune system, including regulation of IFN-γ production, and this could be of importance in both CLE and DM [22].

Detection of complement components (C1q, C3, and C4) and immunoglobulins (Ig) at the dermo-epidermal junction (the so-called lupus band) has been used in CLE diagnostics in many decades using the direct immunofluorescence technique [23]. The test has been criticized for its limited specificity and sensitivity [24]. Deposition of MAC within the CLE and lupus nephritis inflammatory infiltrates has been reported earlier [24, 25]. Also, detection of MAC within the endothelium and perifascicular, usually atrophic, muscle fibers is utilized as a distinct diagnostic feature for DM [25]. Proteomic findings indicate that activation and deposition of complement, including the membrane attack complex (MAC), is more abundant in CLE cutaneous inflammatory foci than DM, where intramuscular MAC activation seems to play a more important role [25].

The limitations of this study include a limited number of individuals included per each group (5 cases per group) that were analyzed in the proteomic analysis; however, the actual findings were confirmed in a higher number of cases (CLE (*n* = 13) and DM (*n* = 7) and both PC (*n* = 5) and HC (*n* = 5)). We acknowledge that less stable proteins could possibly be destroyed during the biopsy handling, and those lower abundant could have been missed by mass spectrometry analysis, since IL-16 was the only cytokine that was abundantly detected by this technique. As we only investigated the inflammatory infiltrates, resident cells located outside of the foci could also be of importance, but might be missed in our proteomic investigation. However, cells expressing IL-16 and caspase-3 could be evaluated by IHC and detected in all three subject groups investigated.

## Conclusion

In conclusion, using a novel technique laser capture microdissection combined with unbiased mass spectrometry investigation, we identified that IL-16 is abundant and the only detectable cytokine in inflammatory foci of CLE lesions. We confirmed high expression of IL-16 by IHC, and also detected focal expression of caspase-3, the enzyme that cleaves IL-16 into its active forms. Abundant deposition of the components of the classical complement activation pathway is another feature of CLE, while abundant expression of IFN regulated proteins is a characteristic of both CLE and DM. Our findings could be useful in diagnosing and differentiating CLE and DM in difficult cases, if validated clinically. These observations offer novel information on molecules involved in disease mechanisms and propose a novel pathway to be explored in the search of CLE therapeutic targets.

## Supplementary Information

**Additional file 1: Figure S1.** Top, overview picture of IL-16 expression in cutaneous lupus erythematosus CLE (x4); below, microphotograph of deep infiltrate in CLE, stained for of IL-16, caspase-3 and isotype control (x20).

**Additional file 2: Table S1.** Characteristics of the cohort: presence of the autoantibodies and information on medications

## Data Availability

The datasets generated and analyzed during the current study are not publicly available due to ethical permit which does not allow data sharing to the third party but are available from the corresponding author on reasonable request, for non-commercial purposes.

## Abbreviations

CLE: Cutaneous lupus erythematosus
DM: Dermatomyositis
SLE: Systemic lupus erythematosus
IL: Interleukin
PC: CLE patient control skin
HC: Healthy control
IHC: Immunohistochemistry
C: Complement
MAC: Membrane attack complex
IQR: Interquartile ratio
CLASI: Cutaneous Lupus Disease Area and Severity Index
CDASI: Cutaneous Dermatomyositis Disease Area and Severity Index
TAP-1: Transporter associated with antigen processing 1
TAP-2: Transporter associated with antigen processing 2
TAPBP: TAP-binding protein
ARS: Aminoacyl-tRNA synthetases
IFN: Interferon
IRP: Interferon-regulated proteins
CNS: Central nervous system
M: Mean
SD: Standard deviation
