# Supplementary figures and images for: Proteome study of cutaneous lupus erythematosus (CLE) and dermatomyositis skin lesions reveals IL-16 is differentially upregulated in CLE
Source: Arthritis Res Ther. 2021 Apr 30;23:132. doi: 10.1186/s13075-021-02511-0 (PMC8086067; doi:10.1186/s13075-021-02511-0)

IL-16

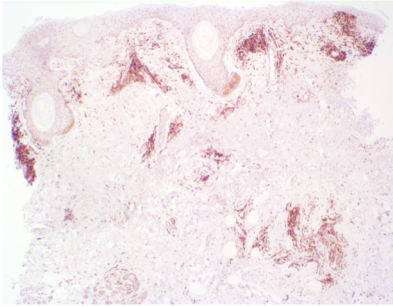

IL-16

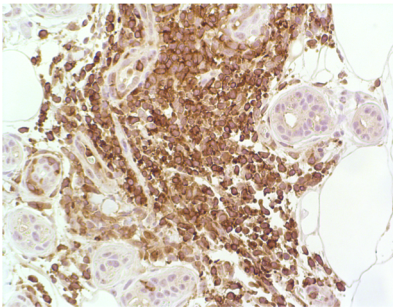

Caspase-3

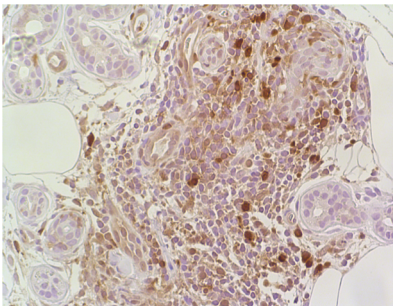

Isotype control

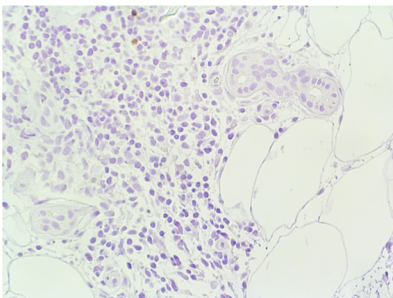

Supplement: Supplementary file 1 — Additional file 1: Figure S1. Top, overview picture of IL-16 expression in cutaneous lupus erythematosus CLE (x4); below, microphotograph of deep infiltrate in CLE, stained for of IL-16, caspase-3 and isotype control (x20). [file 13075_2021_2511_MOESM1_ESM.pdf]
