# Supplementary material for: Proteome study of cutaneous lupus erythematosus (CLE) and dermatomyositis skin lesions reveals IL-16 is differentially upregulated in CLE
Source: Arthritis Res Ther. 2021 Apr 30;23:132. doi: 10.1186/s13075-021-02511-0 (PMC8086067; doi:10.1186/s13075-021-02511-0)
Supplement: Supplementary file 2 — Additional file 2: Table S1. Characteristics of the cohort: presence of the autoantibodies and information on medications [file 13075_2021_2511_MOESM2_ESM.docx]

**Additional Table 1. Characteristics of the cohort: presence of the autoantibodies and information on medications:**

**Autoantibodies CLE DM Healthy CLE**

**controls uninvolved**

n=13 n=7 n=5 n=5

ANA 7 2 nd

anti–SSA/Ro52 7 0 nd

anti–SSA/Ro60 9 0 nd

anti-SmRNP 4 0 nd

anti-dsDNA 0 0 nd

anti-SSB 0 0 nd

anti-Jo1 0 1 nd

anti-TIF 1 0 4 nd

anti-SAE-1 0 1 nd

anti-SRP 0 1 nd

anti-MDA5 0 1 nd

anti-Mi2 0 1 nd

**Medication**

Antimalaria 9 un

Methotrexate 2 un

Cellcept 3 un

Azathioprine 1 un

On steroids 6 6

CLE – cutaneous lupus erythematosus, DM- dermatomyositis, nd – not done, un- information unavailable.

**Additional Table 2. The ratios of the proteins detected by mass-spectrometry analysis.**

Protein ratio CLE/HC DM/HC CLE/DM

2 fold 333 110 246

5 fold 147 47 67

10 fold 76 24 23

Protein ratio HC/CLE HC/DM DM/CLE

2 fold 151 77 90

5 fold 40 18 14

10 fold 17 4 4

HC- healthy controls, CLE – cutaneous lupus erythematosus, DM - dermatomyositis
